# Preoperative ternary classification using DCE-MRI radiomics and machine learning for HCC, ICC, and HIPT

**DOI:** 10.1186/s13244-025-02062-x

**Published:** 2025-08-14

**Authors:** Peng Xie, Zhong-Jian Liao, Lu Xie, Junyuan Zhong, Xiaodong Zhang, Wei Yuan, Yujin Yin, Tianxian Chen, Huizhen Lv, Xinglin Wen, Xiaochun Wang, Ling Zhang

**Affiliations:** 1 Ganzhou Institute of Medical Imaging, Ganzhou Key Laboratory of Medical Imaging and Artificial Intelligence, Department of Medical Imaging, Ganzhou People’s Hospital, Ganzhou Hospital-Nanfang Hospital, Southern Medical University, Ganzhou, China; 2 Gannan Medical University, Ganzhou, China; 3 Department of Nuclear Medicine, Ganzhou People’s Hospital, Ganzhou, China; 4 Fuzhou Medical College of Nanchang University, Fuzhou, China; 5 Department of Radiology, Nanfang Hospital, Southern Medical University, Guangzhou, China

**Keywords:** Hepatocellular carcinoma, Intrahepatic cholangiocarcinoma, Inflammatory pseudotumor, Radiomics, Machine learning

## Abstract

**Objectives:**

This study develops a machine learning model using dynamic contrast-enhanced magnetic resonance imaging (DCE-MRI) radiomics and clinical data to preoperatively differentiate hepatocellular carcinoma (HCC), intrahepatic cholangiocarcinoma (ICC), and hepatic inflammatory pseudotumor (HIPT), addressing limitations of conventional diagnostics.

**Materials and methods:**

This retrospective study included 280 patients (HCC = 160, ICC = 80, HIPT = 40) who underwent DCE-MRI from 2008 to 2024 at three hospitals. Radiomics features and clinical data were extracted and analyzed using LASSO regression and machine learning algorithms (Logistic Regression, Random Forest, and Extreme Gradient Boosting), with class weighting (HCC:ICC:HIPT = 1:2:4) to address class imbalance. Models were compared using macro-average Area Under the Curve (AUC), accuracy, recall, and precision.

**Results:**

The fusion model, integrating radiomics and clinical features, achieved an AUC of 0.933 (95% CI: 0.91–0.95) and 84.5% accuracy, outperforming radiomics-only (AUC = 0.856, 72.6%) and clinical-only (AUC = 0.795, 66.7%) models (*p* < 0.05). Rim enhancement is a key model feature for distinguishing HCC from ICC and HIPT, while hepatic lobe atrophy distinguishes ICC and HIPT from HCC.

**Conclusion:**

This study developed a novel preoperative imaging-based model to differentiate HCC, ICC, and HIPT. The fusion model performed exceptionally well, demonstrating superior accuracy in ICC identification, significantly outperforming traditional diagnostic methods (e.g., radiology and biomarkers) and single-modality machine learning models (*p* < 0.05). This noninvasive approach enhances diagnostic precision and supports personalized treatment planning in liver disease management.

**Critical relevance statement:**

This study develops a novel preoperative imaging-based machine learning model to differentiate hepatocellular carcinoma (HCC), intrahepatic cholangiocarcinoma (ICC), and hepatic inflammatory pseudotumor (HIPT), improving diagnostic accuracy and advancing personalized treatment strategies in clinical radiology.

**Key Points:**

- A machine learning model integrates DCE-MRI radiomics and clinical data for liver lesion differentiation.
- The fusion model outperforms single-modality models with 0.933 AUC and 84.5% accuracy.
- This model provides a noninvasive, reliable tool for personalized liver disease diagnosis and treatment planning.

**Graphical Abstract:**

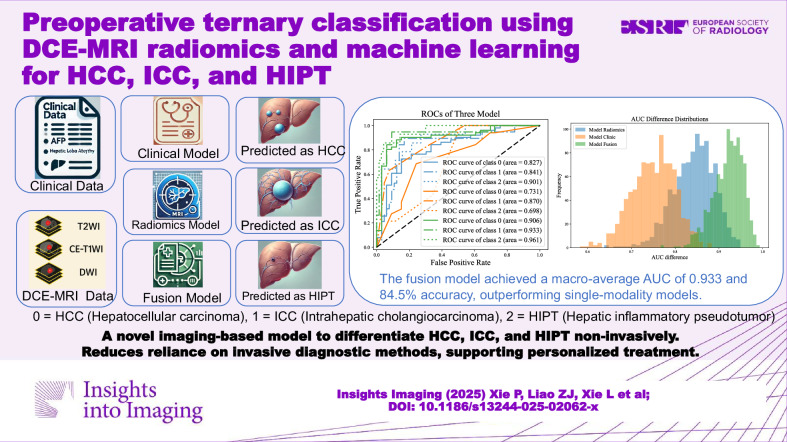

## Introduction

Hepatocellular carcinoma (HCC), intrahepatic cholangiocarcinoma (ICC), and hepatic inflammatory pseudotumor (HIPT) are three liver lesions that present significant diagnostic challenges. HCC accounts for 75–85% of primary liver cancers, ICC constitutes 10–15% with a rising incidence, and HIPT is a rare benign lesion with a prevalence of less than 1% [1–3]. Although HIPT is rare, its imaging characteristics often closely resemble those of HCC and ICC, leading to frequent misdiagnosis as malignant tumors [4, 5]. Accurate preoperative differentiation among these three conditions is essential for determining appropriate treatment strategies. While HCC and ICC typically require surgical or pharmacological interventions, HIPT, being a benign lesion, only necessitates conservative management [6–8]. Misdiagnosis not only results in unnecessary biopsies or surgeries but also increases the risks of complications such as bleeding, infection, and needle tract seeding [9, 10]. Conventional imaging modalities and serum markers have demonstrated limited effectiveness in distinguishing these lesions, exhibiting insufficient sensitivity and specificity [11–16]. Moreover, research on HIPT is limited, and effective models for targeted noninvasive preoperative differentiation of HCC, ICC, and HIPT are currently lacking. Radiomics, combined with machine learning, allows for the extraction of image features, offering a novel approach for noninvasive diagnosis [17].

In this study, we incorporate HIPT into a novel three-way classification framework alongside HCC and ICC. By leveraging MR radiomics and machine learning techniques, we aim to develop a preoperative noninvasive method to differentiate these three entities, with the goal of improving diagnostic accuracy, ultimately providing a scientific basis for individualized treatment.

## Materials and methods

### Study population

This retrospective study included 280 patients from three hospitals who underwent preoperative dynamic contrast-enhanced MRI (DCE-MRI) between May 2008 and January 2024. All patients were pathologically confirmed to have hepatocellular carcinoma (HCC), intrahepatic cholangiocarcinoma (ICC), or hepatic inflammatory pseudotumor (HIPT). The cohort consisted of 160 HCC cases, 80 ICC cases, and 40 HIPT cases. Given the higher prevalence of HCC, 160 eligible HCC cases were randomly selected to match the sample size of ICC and HIPT groups. The study received approval from the Ethics Committee of Ganzhou People’s Hospital (Approval No. PJB2025-249-01), and informed consent was waived.

### Inclusion criteria

1. Age between 18 and 80 years.
2. Pathological confirmation of HCC, ICC, or HIPT.
3. MRI performed within 1 month prior to surgery without any prior adjuvant therapies (e.g., chemotherapy, radiotherapy) or biopsy.
4. Complete tumor resection.

### Exclusion criteria

1. Recurrent liver cancer or multiple malignancies.
2. Poor MRI quality (e.g., significant motion artifacts, inadequate contrast enhancement, or absence of essential sequences hindering lesion assessment) or incomplete enhanced scanning phases.
3. Incomplete clinical or imaging data.

### Clinical and imaging data collection

Clinical and pathological data collected included gender, age, history of hepatitis B virus (HBV) infection, presence of cirrhosis, levels of alpha-fetoprotein (AFP), carcinoembryonic antigen (CEA), and carbohydrate antigen 19-9 (CA19-9). Imaging features assessed encompassed tumor size, lesion count, capsule presence, capsule depression, margin characteristics, shape, bile duct dilation, lipid content, liver lobe atrophy, rim enhancement, peritumoral enhancement, targetoid appearance, and portal vein tumor thrombus. All imaging features were assessed based on LI-RADS v2018 criteria. Definitions of all imaging features and MRI example images are available in the Supplementary Material.

### Statistical analysis

Continuous variables were compared using ANOVA for normally distributed data or the Kruskal-Wallis test for non-normally distributed data, and were presented as mean ± SD or median (IQR), respectively. Categorical variables were compared using the chi-square test or Fisher’s exact test and were presented as counts (percentages).

### Image acquisition and feature extraction

#### MRI scanning

MRI was performed using multiple scanners, including Siemens Verio, Skyra 3.0-T models, equipped with body phased-array coils. The imaging protocol included breath-hold T2-weighted half-Fourier acquisition single-shot turbo spin-echo (HASTE) (axial and coronal, TR/TE = 1600/117 ms, slice thickness = 5 mm), axial TSE T2-weighted images (TR/TE = 2000/97 ms, slice thickness = 5 mm), and 3D-VIBE sequences (TR/TE = 6.97/2.39 ms, flip angle 10°, matrix 256 × 192, field of view 350–400 mm, slice thickness = 3 mm). Diffusion-weighted imaging (DWI) was conducted with b-values of 50, 400, and 800 s/mm² (slice thickness = 5 mm, gap = 1.5–2 mm). Detailed scanning parameters for all scanners are provided in the Supplementary Material. For dynamic contrast-enhanced MRI (DCE-MRI), gadopentetate dimeglumine (Magnevist) was administered via the cubital vein at a dose of 0.1 mmol/kg, injected at 2 mL/s, followed by a 20 mL saline flush at the same rate. Imaging phases included non-enhanced, arterial (20–30 s post-injection), portal venous (60–80 s), and delayed (2–5 min) phases.

#### Image preprocessing and radiomics feature extraction

**Fig. 1:**
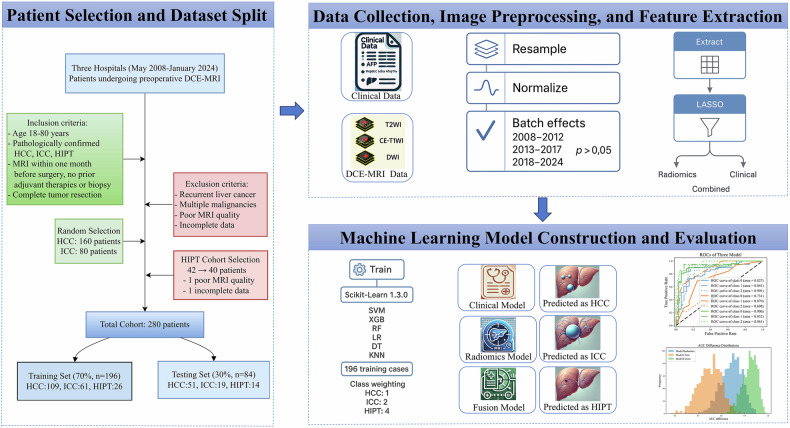
Technical workflow for preoperative ternary classification of HCC, ICC, and HIPT using DCE-MRI radiomics and machine learning

1. **MR image segmentation**: Lesions were manually delineated on T2-weighted, arterial, portal venous, and delayed phase images using ITK-SNAP software. Regions of interest (ROIs) were drawn by a radiologist with 7 years of experience and reviewed by a senior radiologist with 15 years of experience on 50 randomly selected images. Intraclass correlation coefficients (ICCs) were calculated to assess the repeatability of feature extraction, and only features with ICC > 0.9 were included in the analysis.
2. **Radiomics feature extraction**: Using Python 3.10, SimpleITK (2.2.1), and PyRadiomics (3.0.1), radiomic features were extracted from the paired MR images and ROIs. These features included shape, first-order statistics, textural features (GLCM, GLRLM, GLSZM, NGTDM, GLDM), and wavelet-transformed features. Variations in equipment, systems, and acquisition parameters can affect imaging data. To ensure accuracy and stability in subsequent analysis and modeling, raw DICOM images and masks were resampled using linear and nearest-neighbor interpolation, respectively, and pixel intensities were standardized to a 0–1 range via min-max normalization. Additionally, to assess potential batch effects, radiomics features were grouped by acquisition period (2008–2012, 2013–2017, 2018–2024), and ANOVA was performed on key features (e.g., GLCM, GLRLM). No significant inter-group differences were observed (*p* > 0.05, Bonferroni-corrected), so ComBat standardization was not applied.
3. **Feature preprocessing**: Continuous features were standardized using Z-score normalization. LASSO regression was employed to select significant radiomic features from T2-weighted (T2WI), arterial phase (AP), portal venous phase (PVP), and delayed phase (DP) images. Three feature subsets were created for model building: T2WI + AP + PVP + DP radiomics, clinical data, and a combination of T2WI + AP + PVP + DP radiomics with clinical data Fig. 1.

### Machine learning model construction and performance comparison

Six machine learning models from Scikit-Learn (1.3.0) were developed: Support Vector Machine (SVM), Extreme Gradient Boosting (XGB), Random Forest (RF), Logistic Regression (LR), Decision Tree (DT), and K-Nearest Neighbors (KNN). Each feature subset was divided into training (70%) and testing (30%) sets. The dataset (*n* = 280) was randomly split into training (70%, *n* = 196) and test (30%, *n* = 84) sets without class stratification to promote adaptability to clinical heterogeneity. The test set’s class distribution (HCC:ICC:HIPT ≈ 43:17:11) approximated the overall 4:2:1 ratio. Three-class classification models were trained using pathological types as labels. To mitigate the class imbalance (HCC:ICC:HIPT ≈ 4:2:1), we applied class weighting during model training, with weights assigned as HCC:ICC:HIPT = 1:2:4 based on the inverse of class proportions. Five-fold cross-validation and grid search were utilized to identify optimal hyperparameters. Model performance was evaluated on the testing set using metrics such as Area Under the Curve (AUC), accuracy, recall, precision, F1-score, and Kappa.

The optimal models for each feature subset were further validated using the bootstrap method. For each optimal model, 1000 bootstrap samples of the predicted probability scores on the testing set were generated. AUC values were calculated for the three models on these datasets, resulting in AUC distributions for each model.

Performance comparison was conducted by plotting histograms of the AUC distributions. *T*-tests were performed to assess the statistical significance of differences between the models’ AUC distributions.

## Results

### Clinical characteristics

Table 1 demonstrates that there were statistically significant differences in capsule presence, shape, and hepatic lobe atrophy among the hepatocellular carcinoma (HCC), intrahepatic cholangiocarcinoma (ICC), and inflammatory pseudotumor (IP) groups (*p* < 0.05).

**Table 1:** Comparison of baseline characteristics among HCC, ICC, and IP HCC (hepatocellular carcinoma), ICC (intrahepatic cholangiocarcinoma), IP (inflammatory pseudotumor)

	HCC	ICC	IP	Chi/T^a^	*p*-value^a^	Chi/T^b^	*p*-value^b^	Chi/T^c^	*p*-value^c^
Sex				16.547	< 0.001*	2.735	0.098	1.572	0.210
Male	137 (85.6%)	50 (62.5%)	31 (77.5%)						
Female	23 (14.4%)	30 (37.5%)	9 (22.5%)						
Age	54.80 ± 11.829	59.78 ± 9.205	56.70 ± 13.472	−3.578	< 0.001*	1.300	0.199	−0.883	0.378
Size				15.384	< 0.001*	9.075	0.003*	0.305	0.581
< 5 cm	130 (81.3%)	46 (57.5%)	34 (85.0%)						
≥ 5 cm	30 (18.8%)	34 (42.5%)	6 (15.0%)						
AFP				55.778	< 0.001*	0.939	0.625	37.307	< 0.001*
0–20 (μg/L)	70 (43.8%)	75 (93.8%)	39 (97.5%)						
21–400 (μg/L)	66 (41.3%)	4 (5.0%)	1 (2.5%)						
> 400 μg/L	24 (15.0%)	1 (1.3%)	0 (0.0%)						
CEA				11.431	0.001*	7.290	0.007*	1.546	0.214
Negative	154 (96.3%)	67 (83.8%)	40 (100.0%)						
Positive	6 (3.8%)	13 (16.3%)	0 (0.0%)						
CA19-9				30.871	< 0.001*	10.240	0.001*	0.245	0.621
Negative	137 (85.6%)	42 (52.5%)	33 (82.5%)						
Positive	23 (14.4%)	38 (47.5%)	7 (17.5%)						
Cirrhosis				2.283	0.131	2.455	0.117	0.228	0.633
Negative	118 (73.8%)	66 (82.5%)	28 (70.0%)						
Positive	42 (26.3%)	14 (17.5%)	12 (30.0%)						
Hepatitis B				119.213	< 0.001*	2.052	0.152	125.427	< 0.001*
Negative	5 (3.1%)	54 (67.5%)	32 (80.0%)						
Positive	155 (96.9%)	26 (32.5%)	8 (20.0%)						
Lesion count				1.280	0.258	4.865	0.027*	2.198	0.138
Single	148 (92.5%)	77 (96.3%)	34 (85.0%)						
Multiple	12 (7.5%)	3 (3.8%)	6 (15.0%)						
Capsule				5.000	0.025*	15.000	< 0.001*	28.125	< 0.001*
Negative	88 (55.0%)	56 (70.0%)	40 (100.0%)						
Positive	72 (45.5%)	24 (30.0%)	0 (0.0%)						
Rim enhancement				35.270	< 0.001*	0.087	0.768	19.094	< 0.001*
Negative	105 (65.6%)	20 (25.0%)	11 (27.5%)						
Positive	55 (34.4%)	60 (75.0%)	29 (72.5%)						
Peritumoral enhancement				60.181	< 0.001*	2.817	0.093	21.429	< 0.001*
Negative	144 (90.0%)	35 (43.8%)	24 (60.0%)						
Positive	16 (10.0%)	45 (56.3%)	16 (40.0%)						
Margin				30.075	< 0.001*	8.684	0.003*	1.373	0.241
Smooth	104 (65.0%)	22 (27.5%)	22 (55.0%)						
Unsmooth	56 (35.0%)	58 (72.5%)	18 (45.0%)						
Shape				17.134	0.001*	8.083	0.044*	24.832	< 0.001*
Roundness	43 (26.9%)	15 (18.8%)	4 (10.0%)						
Ellipsoid	49 (30.6%)	10 (12.5%)	8 (20.0%)						
Lobulate	45 (28.1%)	31 (38.8%)	8 (20.0%)						
Irregularity	23 (14.4%)	24 (30.0%)	20 (50.0%)						
Lipid				22.574	< 0.001*	#	#	11.728	0.001*
Negative	122 (76.3%)	80 (100.0%)	40 (100.0%)						
Positive	38 (23.8%)	0 (0.0%)	0 (0.0%)						
Hepatic lobe atrophy				17.263	< 0.001*	41.408	< 0.001*	18.027	< 0.001*
Negative	107 (66.9%)	31 (38.8%)	40 (100.0%)						
Positive	53 (33.1%)	49 (61.3%)	0 (0.0%)						
Capsule depression				57.945	< 0.001*	32.591	< 0.001*	2.579	0.108
Negative	143 (89.4%)	35 (43.8%)	39 (97.5%)						
Positive	17 (10.6%)	45 (56.3%)	1 (2.5%)						
Targetoid appearance				35.576	< 0.001*	0.068	0.794	20.851	< 0.001*
Negative	129 (80.6%)	34 (42.5%)	18 (45.0%)						
Positive	31 (19.4%)	46 (57.5%)	22 (55.0%)						
Bile duct dilation				99.453	< 0.001*	22.500	< 0.001*	8.419	0.004*
Negative	158 (98.8%)	36 (45.0%)	36 (90.0%)						
Positive	2 (1.3%)	44 (55.0%)	4 (10.0%)						
Venous thrombus				22.634	< 0.001*	7.925	0.005*	0.505	0.477
Negative	158 (98.8%)	66 (82.5%)	40 (100.0%)						
Positive	2 (1.3%)	14 (17.5%)	0 (0.0%)						

Chi-square test was used except for age, which used *t*-test

*HCC* hepatocellular carcinoma, *ICC* intrahepatic cholangiocarcinoma, *IP* inflammatory pseudotumor

* There was statistical significance between them (*p* < 0.05)

^#^ Both patients were negative, and no statistical difference could be calculated between them

^a^ Comparison of clinical and imaging characteristics between HCC and ICC

^b^ Comparison of clinical and imaging characteristics between ICC and IP

^c^ Comparison of clinical and imaging characteristics between HCC and IP

### Machine learning model performance

**Fig. 2:**
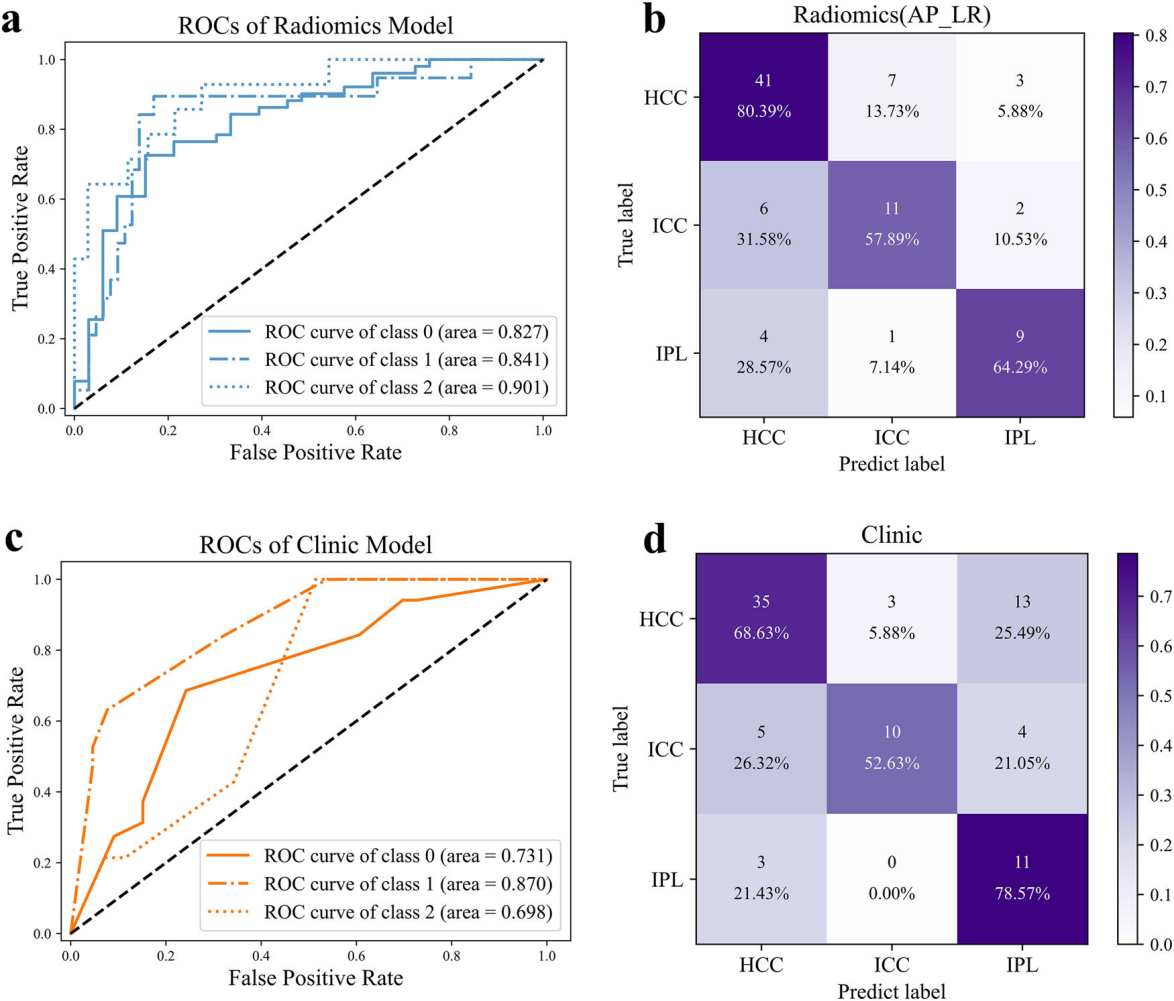
Comparison of classification performance between radiomics-based and clinical feature-based machine learning models. **a** ROC of the radiomics model. **b** Confusion matrix of the radiomics model. **c** ROC of the clinical feature-based model. **d** Confusion matrix of the clinical feature-based model

Among the machine learning models constructed using radiomics features from all four sequences, Logistic Regression (AP sequence) achieved the highest performance, with a macro-average AUC of 0.856, an accuracy of 72.6%, and both recall and precision of 67.5% (Table 2). The ROCs for the radiomics-based models are presented in Fig. 2a. The confusion matrix in Fig. 2b indicates that the Logistic Regression model accurately classified 61 out of 84 cases in the test set (41 HCC, 11 ICC, and 9 IP).

**Table 2:** Comparison of classification performance of the top three machine learning models on the test set

	Accuracy	Precision	Recall	Auc_test	Sensitivity_0	Sensitivity_1	Sensitivity_2	Specificity_0	Specificity_1	Specificity_2
LR_AP	0.726190476	0.675242027	0.675242027	0.856470143	0.803921569	0.578947368	0.642857143	0.696969697	0.876923077	0.928571429
RF_Clinic	0.666666667	0.658680467	0.666101528	0.794592199	0.68627451	0.526315789	0.785714286	0.757575758	0.953846154	0.757142857
LR_AP_CLI	0.845238095	0.813246113	0.841196128	0.933181286	0.843137255	0.894736842	0.785714286	0.878787879	0.907692308	0.957142857

Sensitivity and specificity are reported for each class: 0 = HCC; 1 = ICC; 2 = HIPT

*LR_AP* logistic regression (arterial phase radiomics features), *RF Clinic* Random Forest (clinical features), *LR_AP_CLI* logistic regression (arterial phase radiomics + clinical features), *AUC* area under the curve, *HCC* hepatocellular carcinoma, *ICC* intrahepatic cholangiocarcinoma, *HIPT* hepatic inflammatory pseudotumor

Among the models utilizing clinical features, Random Forest (RF Clinic) exhibited the best performance, with a macro-average AUC of 0.795, an accuracy and recall of 66.7%, and a precision of 65.9% (Table 2). The ROCs for the clinical feature-based models are shown in Fig. 2c. The confusion matrix in Fig. 2d reveals that this model accurately classified 56 cases in total (35 HCC, 10 ICC, and 11 IP). These results suggest that the clinical feature-based model was effective in classifying HCC and IP, but less so for ICC.

**Fig. 3:**
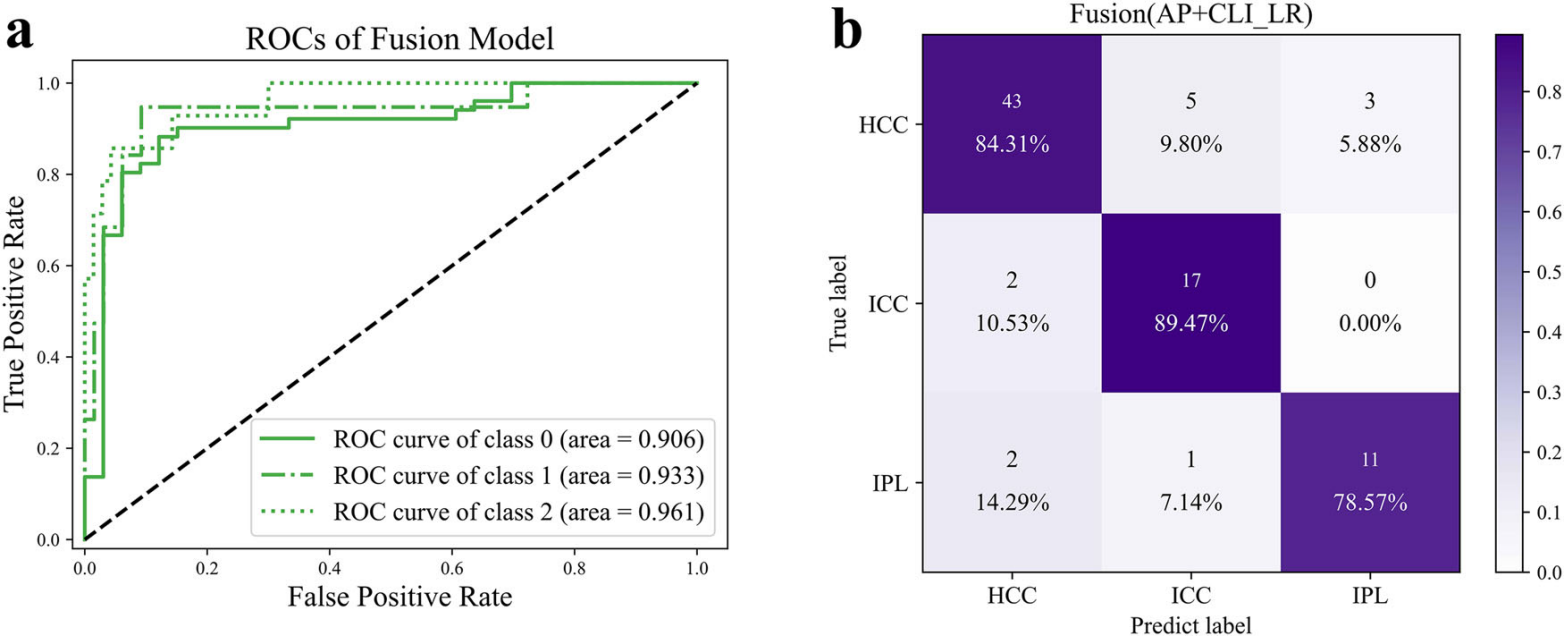
Classification performance of the combined (fusion) model. **a** ROC of the combined model. **b** Confusion matrix of the combined model

Among the fusion models that integrated radiomics features from all sequences with clinical features, the LR_AP_CLI model demonstrated the best performance, achieving a macro-average AUC of 0.933—the highest among all models. This model attained an accuracy of 84.5%, a recall of 84.1%, and a precision of 81.3% (Table 2). The ROCs for the fusion feature-based models are depicted in Fig. 3a. The confusion matrix in Fig. 3b shows that the model accurately classified 71 cases (43 HCC, 17 ICC, and 11 IP). Compared to models based solely on radiomics or clinical features, the fusion model significantly enhanced the accuracy of ICC classification and improved the classification performance for both HCC and IP.

**Fig. 4:**
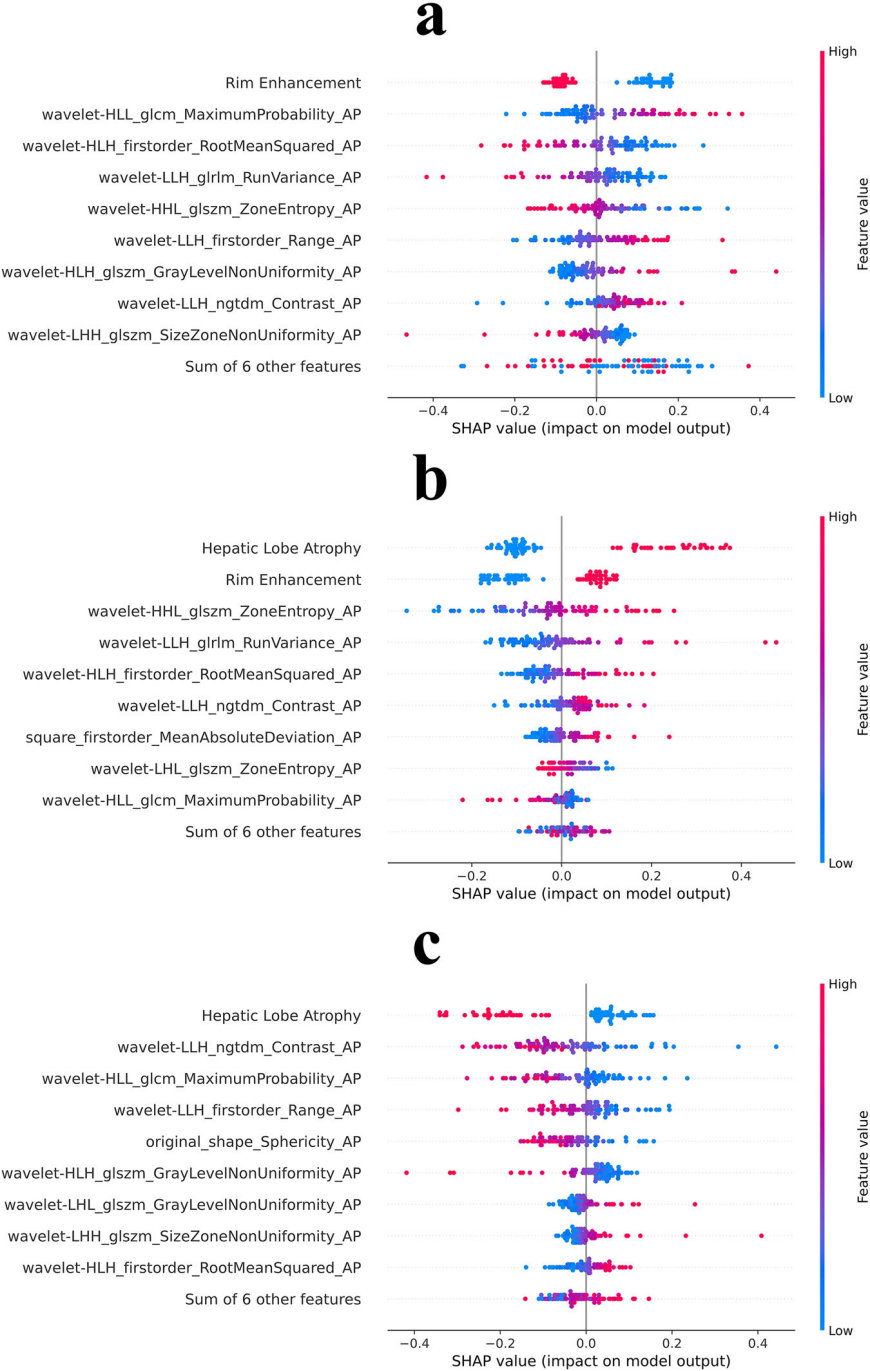
Feature importance analysis for HCC, ICC, and IP. **a** Feature importance for hepatocellular carcinoma (HCC). **b** Feature importance for intrahepatic cholangiocarcinoma (ICC). **c** Feature importance for inflammatory pseudotumor (IP)

Figure 4a illustrates the feature importance analysis for HCC, Fig. 4b for ICC, and Fig. 4c for IP. The five most important features for HCC were rim enhancement, AP sequence GLCM, first-order statistics, GLRLM, and GLSZM features. For ICC, the top features were hepatic lobe atrophy, rim enhancement, AP sequence GLSZM, GLRLM, and first-order features. For IP, the most significant features included hepatic lobe atrophy, AP sequence NGTDM, GLCM, first-order statistics, and original shape features.

**Fig. 5:**
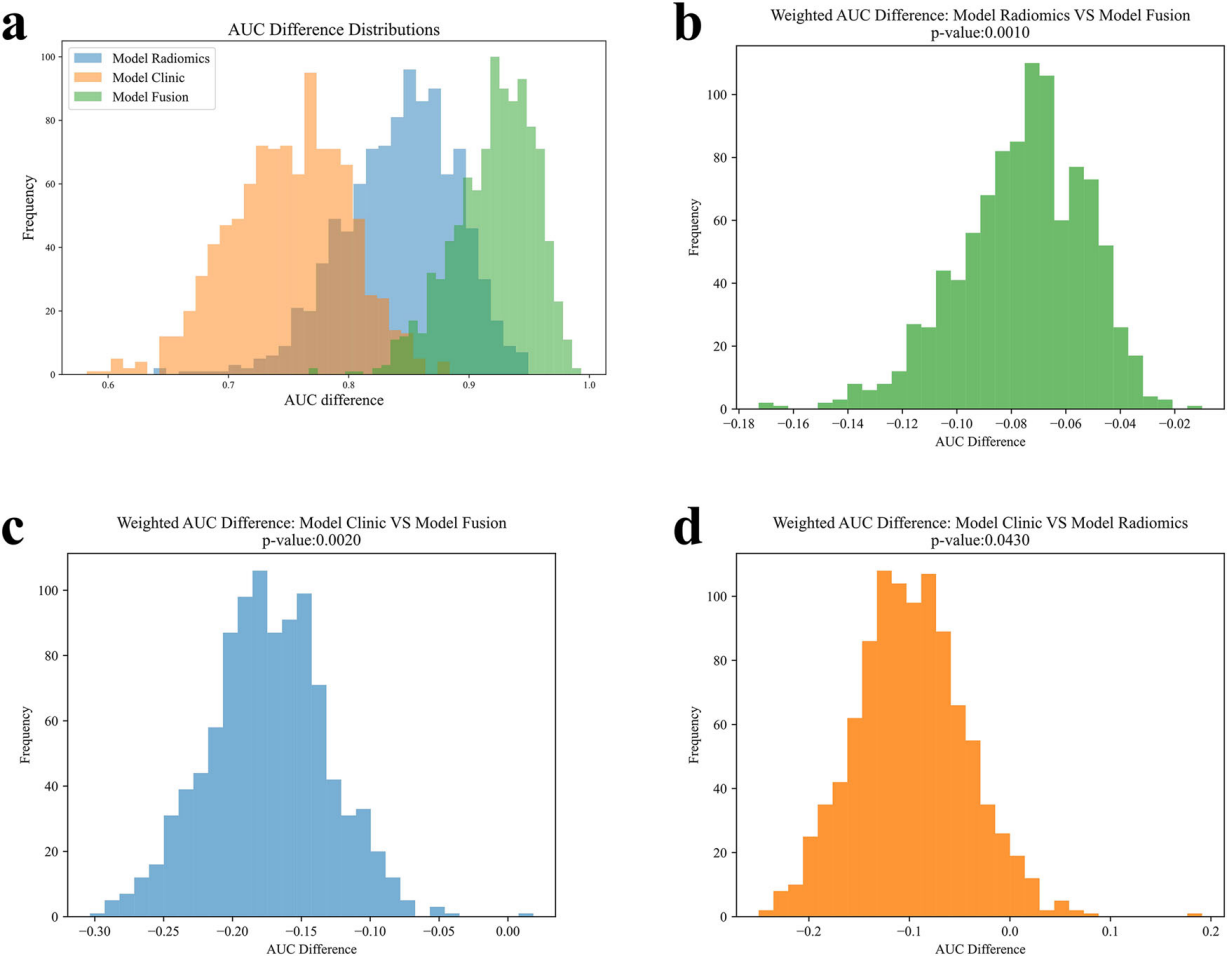
Statistical comparison of macro-average AUCs among different models. **a** Macro-average AUCs for the all-sequence radiomics model, clinical feature model, and fusion model. **b** Comparison of AUC between the all-sequence radiomics model and the fusion model (*p* = 0.001). **c** Comparison of AUC between the clinical feature model and the fusion model (*p* = 0.002). **d** Comparison of AUC between the clinical feature model and the all-sequence radiomics model (*p* = 0.043)

Figure 5a compares the macro-average AUC values of the all-sequence radiomics model, the clinical feature model, and the fusion model, indicating that the fusion model performed the best. Further statistical analysis revealed that the differences in macro-average AUC between the all-sequence radiomics model and the fusion model Fig. 5b, the clinical feature model and the fusion model Fig. 5c, and the clinical feature model and the all-sequence radiomics model Fig. 5d were all statistically significant, with *p*-values of 0.001, 0.002, and 0.043, respectively.

**Fig. 6:**
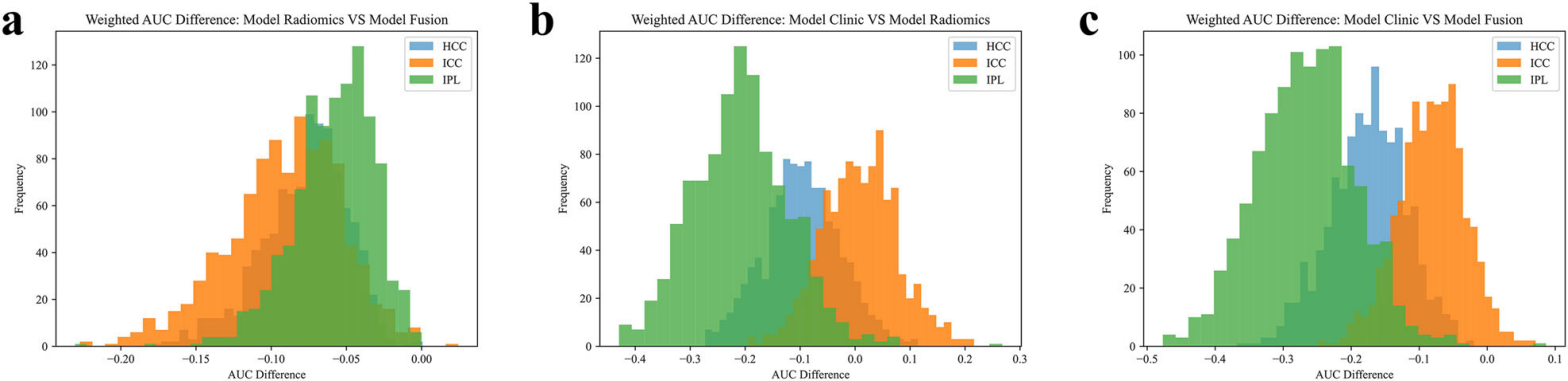
Detailed classification performance of different models across specific tasks. **a**–**c** Performance comparison of the radiomics model, clinical feature-based model, and combined model in classifying HCC, ICC, and IP, respectively

Figure 6 displays the performance of different models in specific classification tasks. The radiomics model and fusion model showed statistically significant differences in classifying HCC, ICC, and IP (*p*-values of 0.002, 0.002, and 0.001, respectively; Fig. 6a). Similarly, the clinical feature model and fusion model exhibited statistically significant differences in classifying HCC, ICC, and IP (*p*-values of 0.001, 0.041, and 0.002, respectively; Fig. 6b). However, there were no statistically significant differences between the clinical feature model and the radiomics model in classifying HCC and ICC (*p*-values of 0.084 and 0.550, respectively; Fig. 6c), although there was a significant difference in classifying IP (*p* = 0.013).

## Discussion

This study introduced a novel fusion machine learning model (LR_AP_CLI) that integrates dynamic contrast-enhanced MRI (DCE-MRI) radiomics features with clinical data to differentiate hepatocellular carcinoma (HCC), intrahepatic cholangiocarcinoma (ICC), and hepatic inflammatory pseudotumor (HIPT). The fusion model demonstrated a macro-average area under the curve (AUC) of 0.933 and an accuracy of 84.5%, significantly surpassing the performance of models based solely on radiomics (AUC = 0.856, accuracy = 72.6%) or clinical features (AUC = 0.795, accuracy = 66.7%). Notably, the model achieved a marked improvement in accurately identifying ICC, addressing the diagnostic challenges associated with traditional imaging modalities.

In comparing our findings with existing literature on diagnostic differentiation of liver lesions, traditional imaging methods show limited sensitivity and specificity in distinguishing subtle features such as tumor morphology, enhancement patterns, and vascular invasion [11–14, 18–20]. A meta-analysis reported MRI achieves a sensitivity of 81% (95% CI 0.79–0.84) and specificity of 90% (95% CI 0.88–0.91) in differentiating ICC from HCC [19]. CEUS demonstrates a sensitivity of 88% (95% CI 0.84–0.90) and specificity of 80% (95% CI 0.78–0.83) [19]. CT yields a sensitivity of 77% and specificity of 86% based on multiphase enhancement [20]. For hepatic inflammatory pseudotumor (HIPT), specific metrics are scarce due to its rarity, with frequent misdiagnosis as malignant tumors requiring biopsy confirmation [4]. These limitations highlight the need for advanced tools. Our fusion model integrating radiomics and clinical data (AUC 0.933) significantly enhances diagnostic differentiation of HCC, ICC, and HIPT, which is critical for guiding precise clinical treatment strategies.

Hepatocellular carcinoma (HCC) remains the most common primary liver malignancy worldwide [1, 21]. In 2022, liver cancer ranked 6th in incidence with 865,269 new cases and 3rd in cancer mortality with 757,948 deaths globally [1]. The global incidence of intrahepatic cholangiocarcinoma (ICC) has been steadily increasing, with a rise of over 140% in the past four decades [22, 23]. In the United States, the incidence of ICC grew at an Annual Percentage Change (APC) of 3.56 from 2001 to 2006, accelerating to 7.67 from 2006 to 2015 [24]. Due to its insidious nature, ICC is often diagnosed at advanced stages, resulting in a poor prognosis [25]. Our model achieves high accuracy (macro-average AUC = 0.933) in distinguishing ICC from other liver lesions, providing robust support for differential diagnosis. Significant differences exist in the treatment strategies for ICC and HCC. HCC is typically managed through liver resection, local treatments such as liver transplantation or radiofrequency ablation, and targeted drug therapy [26]. In contrast, ICC treatment involves not only surgical resection but also lymph node dissection and chemotherapy [27]. Due to a higher propensity for lymph node metastasis, accurate preoperative diagnosis of ICC and appropriate lymph node dissection are critical for significantly improving the postoperative survival rates of stage III ICC patients [28]. On the other hand, the treatment approach for HCC is more straightforward, with current guidelines advising against routine lymph node dissection unless metastasis is confirmed [29]. This necessity for personalized treatment strategies underscores the importance of early diagnosis and targeted therapies in managing both ICC and HCC, emphasizing the need for accurate differentiation among these liver lesions.

In contrast, hepatic inflammatory pseudotumor (HIPT), although a rare benign lesion, often presents with clinical and imaging features similar to liver cancer [30]. This similarity frequently leads to unnecessary invasive procedures, such as biopsy or surgery, increasing the risks of bleeding, infection, and potential tumor cell dissemination, ultimately adversely affecting patient prognosis [4, 5]. Furthermore, existing serum markers have limitations in differentiating these lesions and cannot fully meet clinical needs [15, 16]. Moreover, biopsy, as an invasive procedure, not only increases patient discomfort but may also lead to tumor cell dissemination and intrahepatic metastasis, negatively impacting prognosis [9, 10].

While radiomics has shown promise in disease classification and prognosis prediction for HCC, most prior research has been confined to single-center studies with small sample sizes, focusing primarily on HCC subtypes or differentiating HCC from inflammatory pseudotumors [31–38]. For instance, Liao et al [38] utilized contrast-enhanced ultrasound to differentiate HCC and inflammatory pseudotumors but did not include ICC, thereby limiting its application scope. Additionally, approximately 7% of ICC patients present with a “wash-in and wash-out” enhancement pattern on DCE-MRI, rather than the typical “delayed enhancement” pattern, making ICC prone to misdiagnosis as HCC [39]. Our study uniquely expands this scope by incorporating HIPT into a three-class diagnostic framework alongside HCC and ICC, thereby broadening the diagnostic capabilities and enhancing classification performance. The fusion model’s superior performance in identifying ICC underscores the potential of multimodal feature integration to significantly improve diagnostic accuracy, addressing the challenges posed by traditional imaging methods.

However, this study has several limitations that must be acknowledged. First, the sample size, particularly for HIPT cases, was relatively small, potentially affecting the generalizability of the model. Future research should aim to include larger, multicenter datasets to validate and enhance the model’s robustness. Second, the exclusion of combined hepatocellular-cholangiocarcinoma (cHCC-CC), a subtype that shares imaging characteristics with both HCC and ICC, limits the model’s comprehensive applicability. Incorporating cHCC-CC into future models could improve diagnostic accuracy and completeness. Although this study utilized multiple MRI scanners across three centers, all data were collected within a single country, which may limit the model’s universality. While various scanner types and acquisition parameters were employed (see Supplementary Material), their specific impact on model performance was not systematically analyzed. Future studies incorporating more heterogeneous, multicenter data, potentially including international cohorts, could further validate the model’s generalizability. Additionally, the reliance on manual three-dimensional volumetric segmentation introduces complexity and is time-consuming, which may hinder clinical implementation. Developing automated segmentation tools and integrating additional imaging modalities, such as CT or ultrasound, alongside more extensive clinical variables could address these practical challenges and further optimize the model’s performance and clinical utility. Additionally, the dataset’s 4:2:1 ratio (HCC:ICC:HIPT) diverges from real-world prevalence (approximately 75–85%:10–15%:< 1%) to ensure adequate representation of the clinically significant but rare HIPT class. Class weighting (HCC:ICC:HIPT = 1:2:4) effectively addressed the imbalance, as evidenced by the model’s performance (AUC 0.933). Future studies with larger, prevalence-representative cohorts could further validate the model’s applicability in broader clinical settings.

## Conclusion

This study developed a machine learning model integrating DCE-MRI radiomics and clinical data, achieving a macro-average AUC of 0.933 and accuracy of 84.5% in differentiating HCC, ICC, and HIPT. This model demonstrates high diagnostic accuracy for preoperative identification of these liver lesions, offering potential as a noninvasive diagnostic tool. Further validation in larger, multicenter studies is needed to confirm its clinical utility.

## Supplementary information

ELECTRONIC SUPPLEMENTARY MATERIAL

## Data Availability

The data supporting the results reported in this article can be obtained from the corresponding author upon reasonable request.

## Abbreviations

AFP: Alpha-fetoprotein
AP: Arterial phase
AUC: Area under the curve
CA19-9: Carbohydrate antigen 19-9
CEA: Carcinoembryonic antigen
cHCC-CC: Combined hepatocellular-cholangiocarcinoma
CI: Confidence interval
DCE-MRI: Dynamic contrast-enhanced magnetic resonance imaging
DP: Delayed phase
DT: Decision tree
GLCM: Gray-level co-occurrence matrix
GLOBOCAN: Global Cancer Observatory
GLRLM: Gray-level run-length matrix
GLSZM: Gray-level size zone matrix
HCC: Hepatocellular carcinoma
HIPT: Hepatic inflammatory pseudotumor
ICC: Intrahepatic cholangiocarcinoma
IP: Inflammatory pseudotumor
LASSO: Least absolute shrinkage and selection operator
LR: Logistic regression
MRI: Magnetic resonance imaging
NGTDM: Neighborhood gray-tone difference matrix
PVP: Portal venous phase
RF: Random Forest
ROI: Region of interest
T2WI: T2-weighted imaging
TE: Echo time
TR: Repetition time
