# Supplementary material for: Preoperative ternary classification using DCE-MRI radiomics and machine learning for HCC, ICC, and HIPT
Source: Insights Imaging. 2025 Aug 14;16:178. doi: 10.1186/s13244-025-02062-x (PMC12354431; doi:10.1186/s13244-025-02062-x)
Supplement: Supplementary file 1 — ELECTRONIC SUPPLEMENTARY MATERIAL [file 13244_2025_2062_MOESM1_ESM.pdf]

Preoperative Ternary Classification Using DCE-MRI Radiomics and Machine Learning for HCC, ICC, and HIPT

ELECTRONIC SUPPLEMENTARY MATERIAL

Table of Contents

1. **Appendix 1: Imaging Feature Definitions**
2. **Appendix 2: Example Images**
3. **Appendix 3: Detailed Scanning Parameters**

Appendix 1: Imaging Feature Definitions

This appendix provides definitions for all imaging features assessed in the study, aligned with LI-RADS v2018 criteria where applicable. Example images or diagrams for some features are included in Appendix 2, where available.

1. **Tumor Size**: Maximum diameter of the tumor, measured in millimeters on the clearest MRI sequence/phase (see **Figure S1**).
2. **Lesion Count**: Number of distinct lesions in the liver on MRI, with similar enhancement patterns indicating the same origin.
3. **Capsule Presence**: Smooth, uniform, sharp border surrounding most or all of the lesion, distinctly thicker or more conspicuous than fibrotic tissue around background liver nodules, per LI-RADS v2018. Described as:
   Enhancing "Capsule": A subtype of capsule appearance visible as an enhancing rim in the portal venous phase, delayed phase, or transitional phase on dynamic contrast-enhanced MRI. (see **Figure S2**).
   Nonenhancing "Capsule": A subtype of capsule appearance not visible as an enhancing rim, but as a smooth, uniform, sharp border in the arterial phase, portal venous phase, delayed phase, or transitional phase on dynamic contrast-enhanced MRI; or on unenhanced CT images, unenhanced T1-weighted images, T2-weighted images, T2*-weighted images, diffusion-weighted images, fat fraction maps, or R2* maps. If a border is visible on both enhanced and unenhanced images, it is characterized as an enhancing "capsule". (see **Figure S3**).
4. **Capsule Depression**: Retraction and deformation of the liver capsule due to contractile fibrous stroma within the lesion.
5. **Margin Characteristics**: Appearance of the tumor border, described as smooth (arc-like appearance) or unsmooth (nodular or lobulated protrusion).
6. **Shape**: Overall morphology of the lesion, described as roundness (circular contour), ellipsoid (oval contour), lobulate (lobulated contour), or irregularity (irregular contour) (see **Figure S4**).
7. **Bile Duct Dilation**: Dilatation of intrahepatic bile ducts within or adjacent to the lesion.
8. **Lipid Content**: Presence of intralesional fat, identified by signal drop on opposed-phase T1-weighted images (see **Figure S5**).
9. **Liver Lobe Atrophy**: Volume reduction and morphological distortion of the liver lobe containing or adjacent to the lesion.
10. **Rim Enhancement**: Ring-like enhancement confined to the lesion periphery (see **Figure S6**).
11. **Peritumoral Enhancement**: Irregular patchy or crescent-shaped enhancement in the liver parenchyma surrounding the lesion (see **Figure S7**).
12. **Targetoid Appearance**: A concentric pattern of enhancement or diffusion in the lesion, characterized by one or more of the following features: a rim of hyperenhancement at the periphery of the lesion, a peripheral washout where the outer region shows reduced enhancement compared to the center, a delayed central enhancement where the central area

progressively enhances over time, or a targetoid restriction pattern with restricted diffusion at the periphery and less restricted diffusion in the center (see **Figure S8**).

13. **Portal Vein Tumor Thrombus**: Tumor extension into the portal vein, identified as a filling defect on contrast-enhanced MRI (see **Figure S9**).

Appendix 2: Example Images

This appendix provides example images and schematic diagrams illustrating the imaging features defined in Appendix 1.

- **Figure S1**:

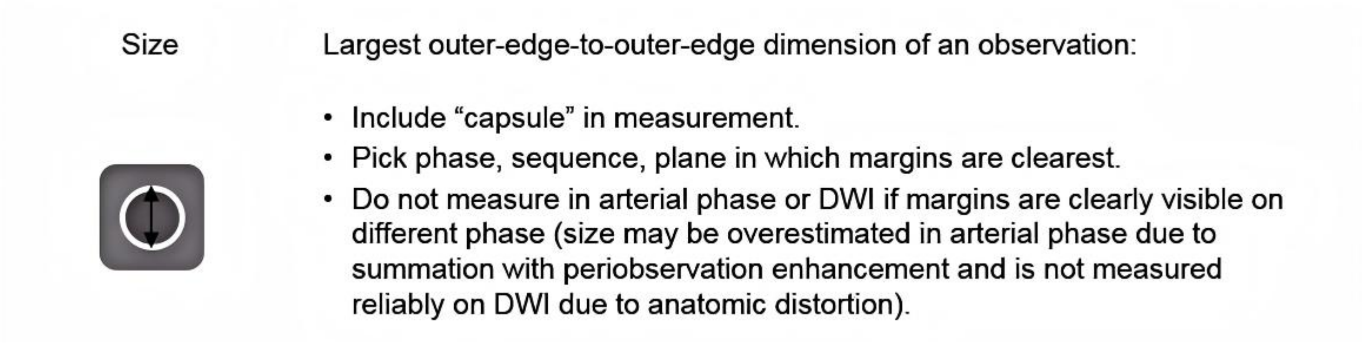

- **Figure S2**: Example of capsule presence.

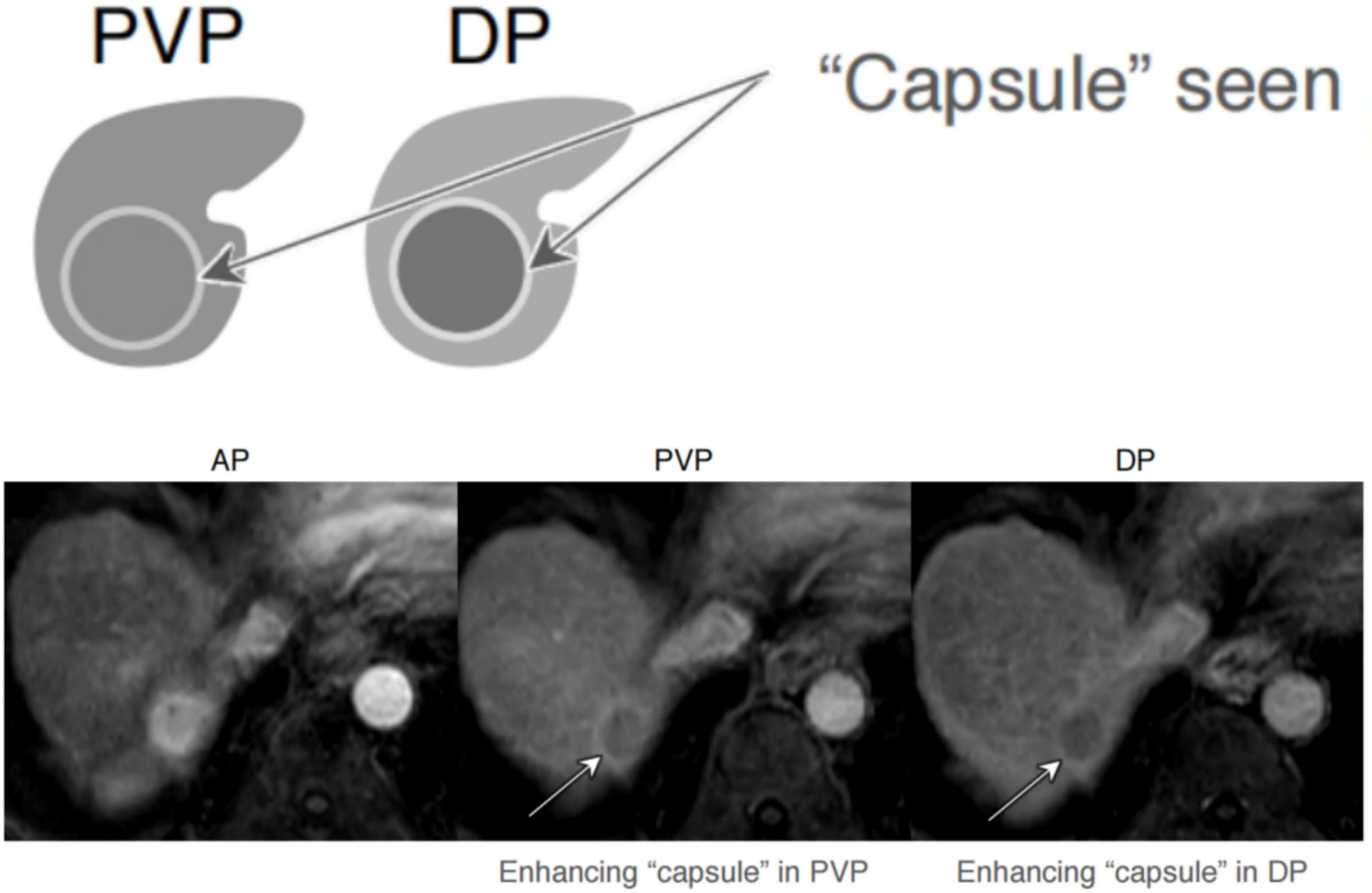

Insights Imaging (2025) Xie P, Liao ZJ, Xie L, et al.

- **Figure S3**:

Nonhancing “capsule” is present if:

- There is a smooth, uniform, sharp border around most or all of an observation, unequivocally thicker or more conspicuous than fibrotic tissue around background nodules on one or more of the phases or sequences described above.

AND

- The rim does not enhance. If the rim enhances progressively, it should be characterized as enhancing “capsule” (major feature of HCC), not as nonenhancing “capsule” (ancillary feature favoring malignancy).

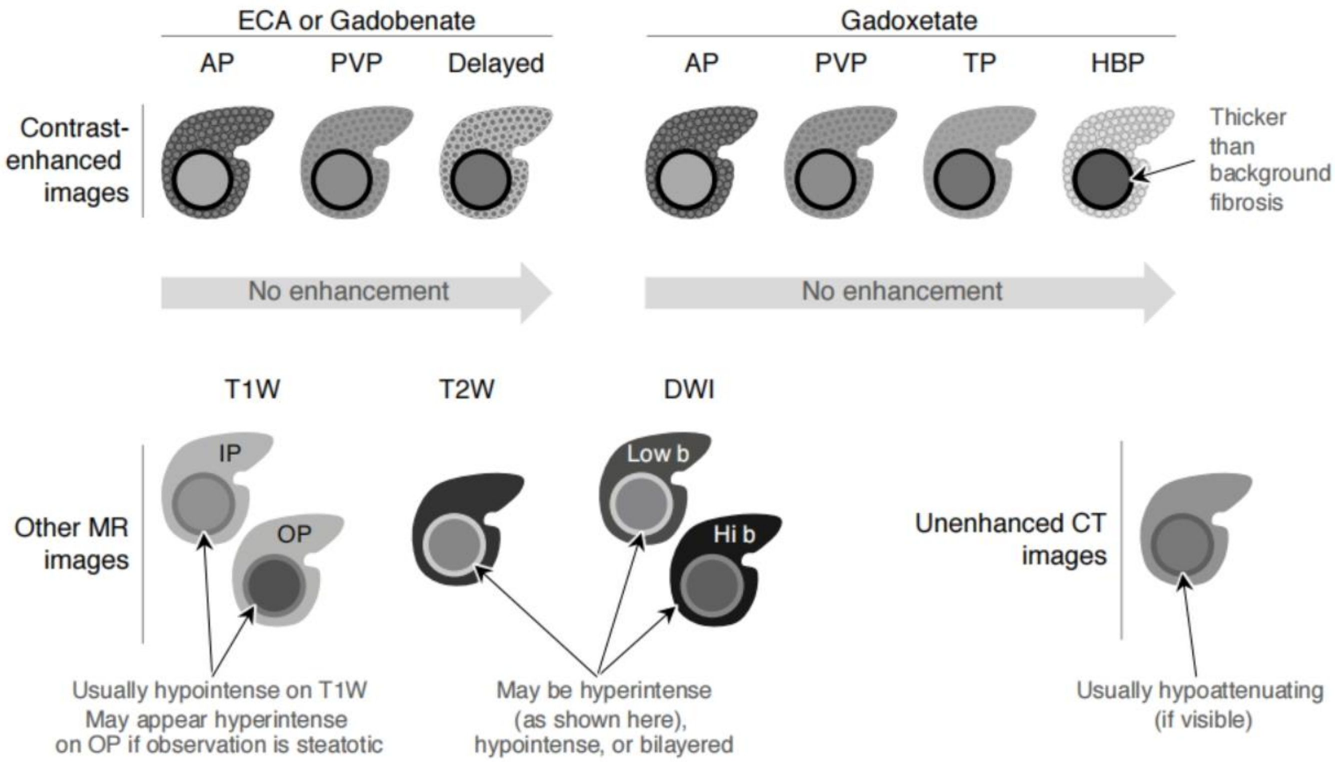

The rim may be visible on only or a small number of phases or sequences. It does not need to be visible on every phase and sequence.

Figure S4: Example of shape.

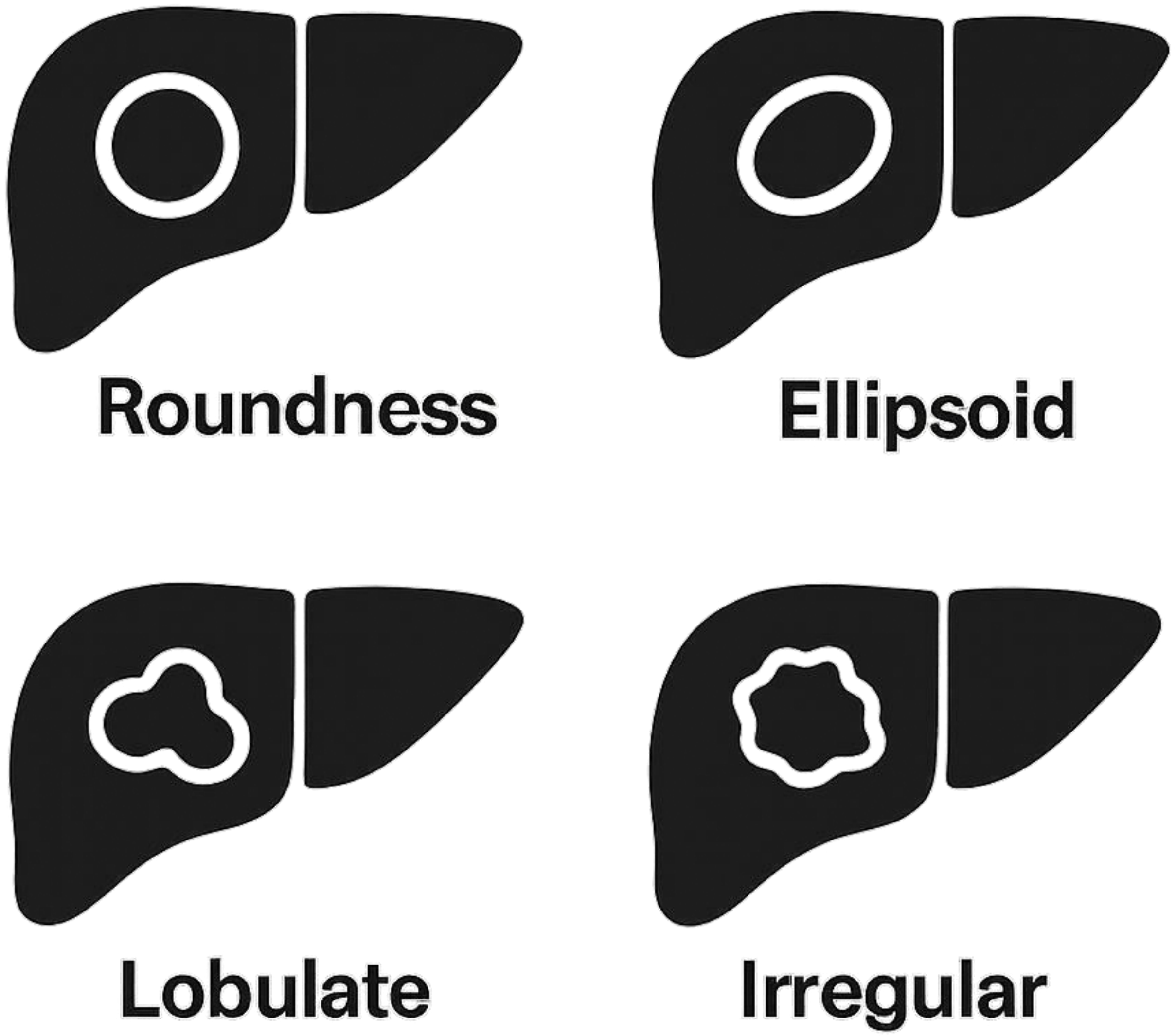

- **Figure S5**: Example of lipid content.

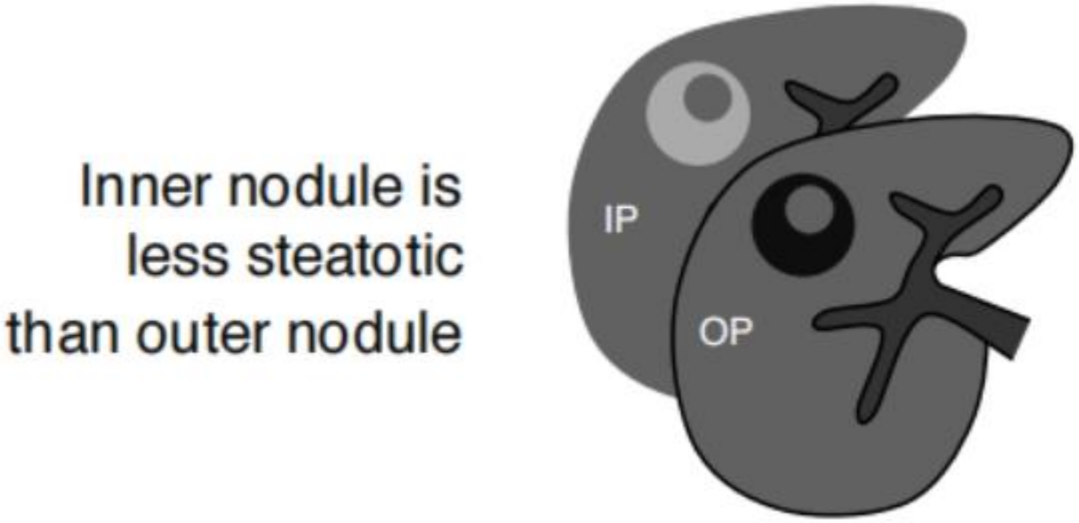

- **Figure S6**: Example of rim enhancement.

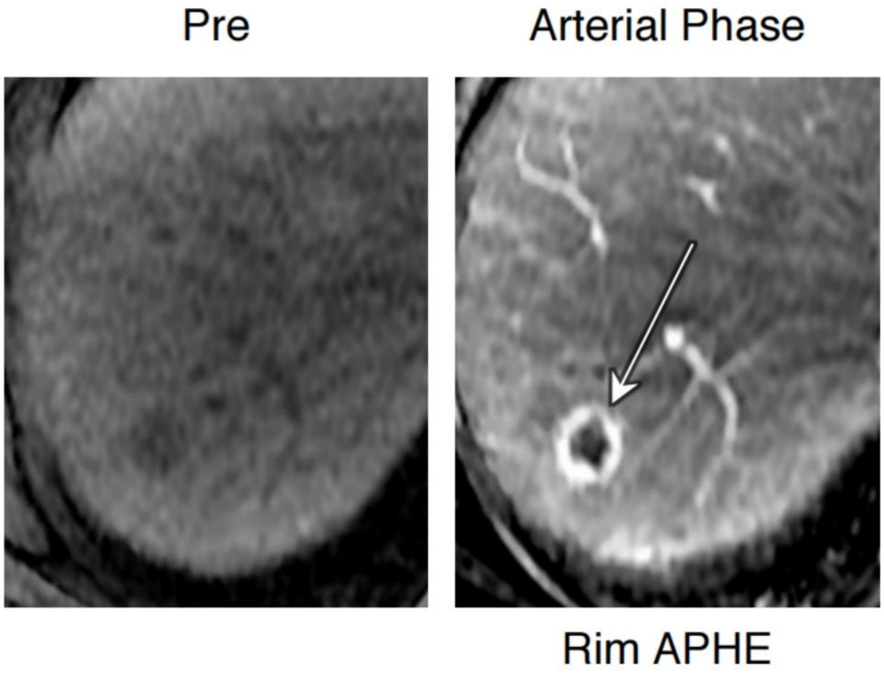

- **Figure S7**: Example of peritumoral enhancement. Arterial-phase MRI showing enhancement in the liver parenchyma surrounding the lesion (arrow).

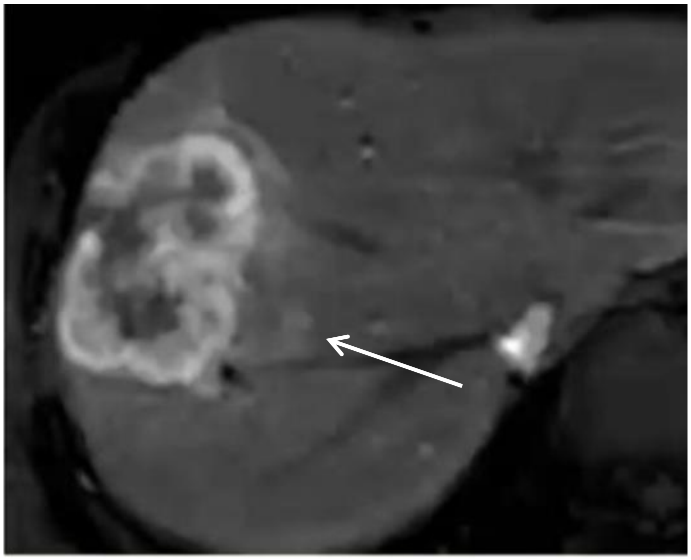

- **Figure S8**:

Targetoid mass, imaging appearance on various phases or sequences

Targetoid dynamic enhancement:

Rim APHE	Spatially defined subtype of APHE in which arterial phase enhancement is most pronounced in observation periphery
Peripheral “washout”	Spatially defined subtype of “washout” in which apparent washout is most pronounced in observation periphery
Delayed central enhancement	Central area of progressive postarterial phase enhancement

Targetoid appearance on DWI or TP/HBP:

Targetoid restriction	Concentric pattern on DWI characterized by restricted diffusion in observation periphery with less restricted diffusion in observation center

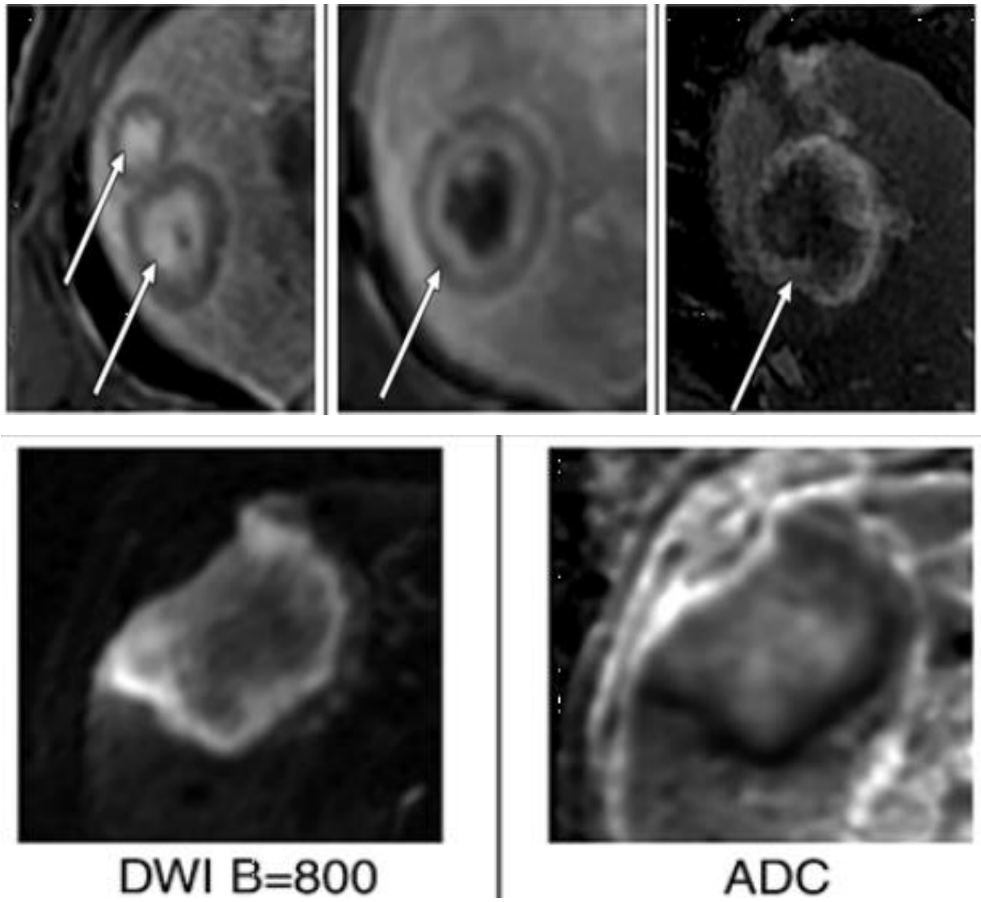

- **Figure S9**: Example of portal vein tumor thrombus.

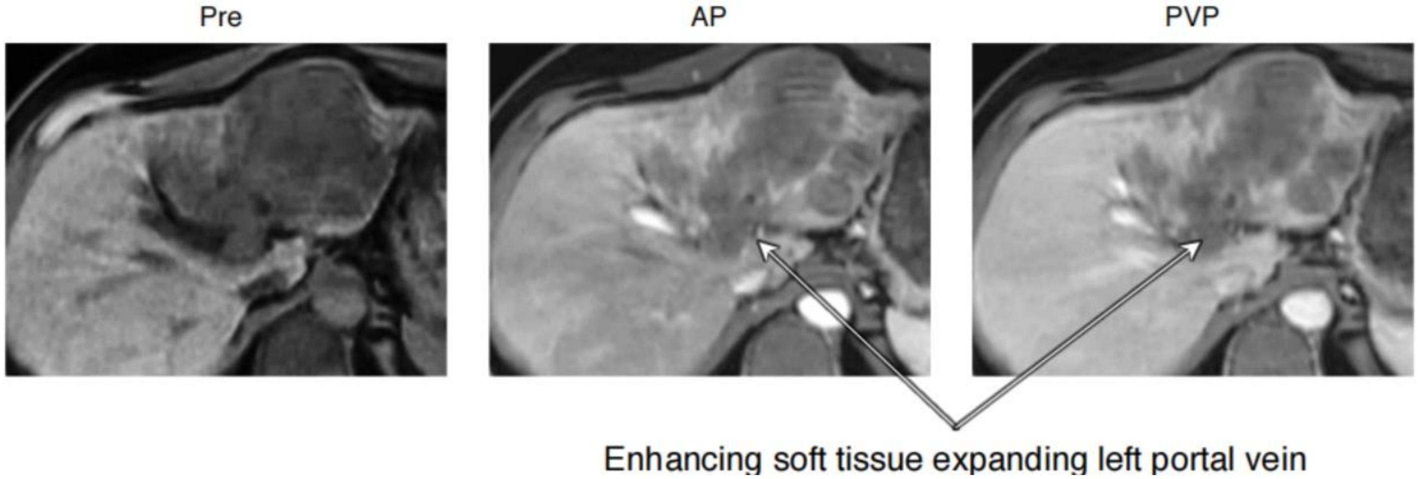

Appendix 3: Detailed Scanning Parameters

This appendix provides detailed scanning parameters for the MRI scanners used in the study, collected from three centers. Data were acquired using Siemens Verio 3.0T, Siemens Skyra 3.0T, GE Optima MR360 1.5T, and GE Signa HDxt 3.0T scanners.

Table S1: Siemens Verio 3.0T and Siemens Skyra 3.0T

Both scanners used body phased-array coils with identical protocols.

Sequence	Type	TR (ms)	TE (ms)	Slice Thickness (mm)	Matrix	FOV (mm)	Flip Angle (°)	Phases/Notes
T2-weighted HASTE (Axial/Coronal)	Single-shot Fast Spin Echo	1600	117	5	256×192	350–400	–	Breath-hold (~20–30 s)
Axial TSE T2-weighted	Turbo Spin Echo	2000	97	5	256×192	350–400	–	Fat suppression optional
3D-VIBE (T1-weighted)	3D Gradient Echo	6.97	2.39	3	256×192	350–400	10	Pre-contrast, Late Arterial (20–30 s), Portal Venous (60–80 s), Delayed (2–5 min)
DWI	Echo Planar Imaging	5000	70	5	128×128	350–400	–	b-values: 50, 400, 800 s/mm²; Slice gap: 1.5–2 mm

Table S2: GE Optima MR360 1.5T

Equipped with a 16-channel abdominal coil.

Sequence	Type	TR (ms)	TE (ms)	Slice Thickness (mm)	Matrix	FOV (mm)	Flip Angle (°)	Phases/Notes
T2-weighted SSFSE (Axial/Coronal)	Single-shot Fast Spin Echo	1000	80	5	320×192	400×352	–	Breath-hold, fast acquisition
Axial FSE T2-weighted	Fast Spin Echo	3600	90	5	320×192	400×352	–	Slice spacing: 5 mm; Fat suppression optional
LAVA (T1-weighted)	3D Gradient Echo	3.8	1.7	2.0	320×192	400×352	12	Pre-contrast, Late Arterial (20–30 s), Portal Venous (60–80 s), Delayed (2–5 min)
DWI	Echo Planar Imaging	5000	70	5	128×128	400×352	–	b-values: 50, 1000 s/mm²

Table S3: GE Signa HDxt 3.0T

Equipped with a 16-channel abdominal coil.

Sequence	Type	TR (ms)	TE (ms)	Slice Thickness (mm)	Matrix	FOV (mm)	Flip Angle (°)	Phases/Notes
T2-weighted SSFSE (Axial/Coronal)	Single-shot Fast Spin Echo	1000	80	5	320×224	420×420	–	Breath-hold, fast acquisition
Axial FSE T2-weighted	Fast Spin Echo	5100	85	5	320×224	420×420	–	Slice spacing: 5 mm; Fat suppression optional
LAVA (T1-weighted)	3D Gradient Echo	5.0	1.7	2.5	320×224	420×420	12	Pre-contrast, Late Arterial (20–30 s), Portal Venous (60–80 s), Delayed (2–5 min)

Sequence	Type	TR (ms)	TE (ms)	Slice Thickness (mm)	Matrix	FOV (mm)	Flip Angle (°)	Phases/Notes
DWI	Echo Planar Imaging	5000	70	5	128×128	420×420	–	b-values: 50, 1000 s/mm²

Abbreviations

The following abbreviations are used in this Electronic Supplementary Material:

- **3D-VIBE: Three-Dimensional Volumetric Interpolated Breath-hold Examination**
- **AP: Arterial Phase**
- **APHE: Arterial Phase Hyperenhancement**
- **CT: Computed Tomography**
- **DP: Delayed Phase**
- **DCE-MRI: Dynamic Contrast-Enhanced Magnetic Resonance Imaging**
- **DWI: Diffusion-Weighted Imaging**
- **FOV: Field of View**
- **FSE: Fast Spin Echo**
- **HASTE: Half-Fourier Acquisition Single-shot Turbo spin Echo**
- **HBP: Hepatobiliary Phase**
- **HCC: Hepatocellular Carcinoma**
- **HIPT: Hepatic Inflammatory Pseudotumor**
- **ICC: Intrahepatic Cholangiocarcinoma**
- **IP: In-Phase (T1-weighted)**
- **LAVA: Liver Acquisition with Volume Acceleration**
- **LI-RADS: Liver Imaging Reporting and Data System**
- **MRI: Magnetic Resonance Imaging**
- **OP: Opposed-Phase (Out-of-Phase)**
- **PVP: Portal Venous Phase**
- **Pre: Pre-contrast**
- **R2*: Apparent Transverse Relaxation Rate**
- **SSFSE: Single-Shot Fast Spin Echo**
- **TE: Echo Time**
- **TR: Repetition Time**
- **T2*: Effective Transverse Relaxation Time**
- **TSE: Turbo Spin Echo**
